# Control of Intermale Aggression by Medial Prefrontal Cortex Activation in the Mouse

**DOI:** 10.1371/journal.pone.0094657

**Published:** 2014-04-16

**Authors:** Aki Takahashi, Kazuki Nagayasu, Naoya Nishitani, Shuji Kaneko, Tsuyoshi Koide

**Affiliations:** 1 Mouse Genomics Resource Laboratory, National Institute of Genetics (NIG), Mishima, Shizuoka, Japan; 2 Department of Genetics, The Graduate University for Advanced Studies (SOKENDAI), Mishima, Shizuoka, Japan; 3 Laboratory of Molecular Neuropharmacology, Graduate School of Pharmaceutical Sciences, Osaka University, Suita, Osaka, Japan; 4 Department of Molecular Pharmacology, Graduate School of Pharmaceutical Sciences, Kyoto University, Kyoto, Kyoto, Japan; University of Medicine & Dentistry of NJ - New Jersey Medical School, United States of America

## Abstract

Aggressive behavior is widely observed throughout the animal kingdom because of its adaptiveness for social animals. However, when aggressive behavior exceeds the species-typical level, it is no longer adaptive, so there should be a mechanism to control excessive aggression to keep it within the adaptive range. Using optogenetics, we demonstrate that activation of excitatory neurons in the medial prefrontal cortex (mPFC), but not the orbitofrontal cortex (OFC), inhibits inter-male aggression in mice. At the same time, optogenetic silencing of mPFC neurons causes an escalation of aggressive behavior both quantitatively and qualitatively. Activation of the mPFC suppresses aggressive bursts and reduces the intensity of aggressive behavior, but does not change the duration of the aggressive bursts. Our findings suggest that mPFC activity has an inhibitory role in the initiation and execution, but not the termination, of aggressive behavior, and maintains such behavior within the adaptive range.

## Introduction

The prefrontal cortex has been implicated in the inhibitory control of emotional outbursts, including aggression and violence [1], [2]. It sends glutamatergic projections to several of the brain areas linked to aggression, such as the hypothalamus, amygdala, periaqueductal gray and dorsal raphe nucleus (DRN) [3]–[5]. In humans, lesions to the prefrontal cortex, especially the OFC and the mPFC, have been shown to cause the onset of impulsive and antisocial behaviors, and the activity of the prefrontal cortex was also found to be decreased in violent patients compared with the level in controls [2], [6]–[8]. Similar to the case in humans, bilateral lesions of either OFC or mPFC increases inter-male and shock-induced aggression in the rat [9], [10]. Aggressive encounters induce the expression of c-Fos, a marker of neural activation, in the OFC and mPFC of the rat and mouse [11]–[14]. Interestingly, this activation of the mPFC induced by an aggressive encounter was blunted in an animal model of escalated aggression in which rats were socially isolated for 4 weeks [13]. Thus, the prefrontal cortex seems to have an inhibitory role in aggressive behavior, in order to maintain it at the species-typical (adaptive) level and prevent it from reaching a maladaptive level. However, these previous studies lacked the temporal resolution to allow the determination of whether the activations of the prefrontal cortex areas are involved in the initiation, execution or termination of aggressive behaviors. Electrical stimulation in cats has shown that activation of the prefrontal cortex area delays the onset of a predatory attack or an affective display induced by hypothalamic stimulation [15], [16], which suggests an inhibitory role in the initiation of aggression in this specific condition. In the present study, we assessed the role of the prefrontal cortex in inter-male aggression in mice. Using optogenetics, we controlled the activation of either mPFC or OFC excitatory neurons to examine the temporal role of prefrontal cortex activity in aggressive behaviors in male mice.

## Materials and Methods

### Animals

Male ICR mice (CLEA Japan, Inc., Tokyo, Japan), aged 5 weeks at the start of the preparations for the experiment, were used as residents and intruders. C57BL/6JJcl mice, purchased from CLEA Japan, Inc., and bred in our animal facility, were also used as intruders (see *Resident–intruder test training*). Each resident male was housed together with a female in a polycarbonate cage (22×32×13.5 cm) with wood chips as bedding material. In total, 38 resident males were used for the aggression test (n = 10 for mPFC–ChETA, n = 9 for mPFC–EYFP, n = 9 for OFC–ChETA, and n = 10 for mPFC–eArchT3.0). Intruder males were housed in groups of five to seven per cage (22×32×13.5 cm) with wood chips as bedding material. All experiments were conducted at the National Institute of Genetics (NIG), Japan, with controlled humidity and temperature (50±10%, 23±2°C) under a 12-h light/dark cycle (room lights off at 6:00 p.m.). Food and water were freely available. All behavioral experiments were performed during the dark period. All procedures were approved by the Committee for Animal Care and Use of the NIG (permit numbers 23-10, 24-10 and 25-10). All surgery was performed under anesthesia, with maximal effort made to minimize suffering.

### Viral production

DNA constructs for CaMKIIα::ChETA-EYFP and CaMKIIα::eArchT3.0-EYFP (pLenti-CaMKIIα-ChETA-EYFP and pLenti-CaMKIIα-eArchT 3.0-EYFP) were obtained from Addgene (Plasmid 26967, Plasmid 35513) as reported previously [17], [18]. The DNA construct for EF1α::EYFP (F46L/F64L/M153T/V163A/S175G; Venus) (pCSII-EF-Venus) was kindly provided by Dr. Hiroyuki Miyoshi (RIKEN BioResource Center, Japan). For the construction of a DNA vector for CaMKIIα::EYFP (pLenti-CaMKIIα-EYFP), the EYFP fragment was amplified from pLenti-CaMKIIα-ChETA-EYFP by PCR using Q5 DNA polymerase (New England Biolabs, Ipswich, USA) and oligodeoxynucleotide primers (Fw: TAC GGA TCC GCC ACC ATG GTG AGC AAG GG, Rv: GCC GAA TTC TTA CTT GTA CAG CTC GTC CA). Amplified EYFP fragments and pLenti-CaMKIIα-ChETA-EYFP were digested with EcoRI and BamHI. Digested EYFP fragments and shuttle vector fragments were purified by agarose gel electrophoresis and gel extraction, and ligated using T4 DNA ligase (BioAcademia, Osaka, Japan). A constructed vector (pLenti-CaMKIIα-EYFP) was verified by sequencing. Lentiviral vectors were essentially prepared as described previously [19], but with some modifications. Briefly, 31.2 µg of pNHP, 12.4 µg of pHEF-VSVG and 15.5 µg of shuttle construct were transfected to Lenti-X 293T cells (Clontech, Mountain View, USA) in 2×15-cm dishes using polyethylenimine (Polyethylenimine “Max”, Mw 40,000; Polysciences, Warrington, USA). After 16–18 hrs of incubation, culture supernatant was collected (first harvest) and fresh media were added. After 30 hrs of incubation, the culture supernatant was collected and mixed with the first harvest. Lentiviral vector-containing media were filtered through a 0.45-µm PVDF filter and ultracentrifuged at 23,000 rpm using an SW-28 rotor (Beckman-Coulter, Inc., Brea, USA) for 2 hrs. After ultracentrifugation the supernatant was removed and the precipitates were resolved in sterile phosphate buffer saline (PBS). Lentiviral vectors were aliquoted and stored at −80°C. The titer of the lentiviral vector solution was estimated by p24 ELISA (BioAcademia) and found to be around 4–5×10^10^ IU/mL.

### Viral infection in mPFC and OFC

Test male was housed with a female for at least 3 weeks before the stereotaxic surgery to enhance territorial aggression. Resident males were anesthetized by i.p. injection of a mixture of 100 mg/kg ketamine HCl and 10 mg/kg xylazine, and were then stereotaxically implanted with a 26-gauge guide cannula (Plastics One Inc., Roanoke, USA) aimed directly above the mPFC (AP, +2.0 mm; ML, +0.3 mm; DV, −1.7 mm to bregma) or the OFC (AP, +2.0 mm; ML, +1.0 mm; DV,−1.7 mm to bregma) as calculated from a mouse brain atlas [20]. A 33-gauge microinjector (Plastics One Inc., Roanoke, USA), which extended 0.3 mm below the end of the guide, was inserted into the guide cannula, and 0.5 µl of viral solution was microinjected over a 5-min period. The microinjector was left in place for 5 min after the infusion to allow the virus to diffuse completely. After surgery, males were housed individually without a female because some of the females bit and broke the guide cannula before the test.

### Resident–intruder test training

One week after surgery, animals were exposed to situations in which aggressive behavior was exhibited using a resident–intruder test every other day. The same male intruder was introduced into the home cage of a resident male every encounter unless the intruder showed attack bites toward the resident male. The behaviors of resident were observed for 5 min after the first bite, or the intruder was removed after 10 min if no attack bite occurred. Once the resident males had showed more than 10 bites for more than three consecutive encounters, we conducted the optical stimulation experiment using that animal. Given that the time required varied between animals to achieve this criterion, the number of encounters for each animals ranged from 6 to 14. Eight resident males did not show any aggressive behavior toward an ICR intruder male, even after several training sessions (more than seven encounters). For those resident males, we used C57BL/6JJcl males as the intruder (2 out of 10 animals in mPFC–ChETA, 2 out of 9 animals in mPFC–EYFP and 4 out of 9 animals in OFC–ChETA); ICR mice show high level of aggression toward C57BL/6 [21]. Indeed, all of those resident males in our study showed aggressive behavior toward C57BL/6JJcl intruders. There was no difference in the effect of optical activation when we used either an ICR intruder or a C57BL/6JJcl intruder.

### In vivo optical stimulation during the aggressive encounter

The optical stimulation test was conducted after animals showed stable aggressive behaviors during resident–intruder training, which was evident 3–6 weeks after viral infection. A 250-µm plastic optical fiber (COME2-DF1-250, Lucir Inc., Tsukuba, Japan) was inserted into the guide cannula (the flat tip of the optic fiber that stops at the end of the guide cannula, which resides directly above the brain target) under isoflurane inhalation anesthesia at least 6 hrs before Trial 1 of the aggression test. The optic fiber was connected to an optical swivel (COME2-UFC, Lucir Inc.), and then connected to a laser light source (COME-2-LY-1, Lucir Inc.) that was controlled by either a schedule stimulator (Lucir Inc.) or a USBpulse100 system (ELAN Digital System Ltd., Hampshire, England). The test male was kept in the test cage (19.2×29×30 cm) with bedding materials moved from his home cage, where food and an agar block (water) were available. After the room lights off (6 p.m.), an intruder male was introduced into the test cage and the animals' behaviors were recorded for 3 min without optical stimulation (Trial 1). At least 3 hrs after Trial 1 (ranging from 3–4.5 hours), we reintroduced the same intruder into the test cage (Trial 2). In Trial 2, optical stimulation was delivered 5 sec before the introduction of the intruder and continued for the first 3 min of the aggressive encounter. For the ChETA experiment, blue light (472 nm) was delivered in 3-ms pulses at 20 Hz, at a final output power of around 5 mW. For the eArchT3.0 experiment, continuous yellow light (589 nm) was delivered at a final output power of around 5 mW. The intruder male was left in the test cage for an additional 3 min after the optical stimulation ceased. Thus, an encounter during Trial 2 lasted a total of 6 min, with the first half involving optical stimulation and the second half not involving any stimulation. Trial 3 was conducted the next day after room lights off, with alternation of the presence or absence of optical stimuli during the 12-min aggressive encounter. Optical stimulation was not delivered for the first 2 min after the intruder was introduced, and then 2 min of 20-Hz (3-msec) stimulation was delivered three times (from 3–4 min, 7–8 min and 11–12 min) with 2-min intervals without stimulation.

### In vivo optic stimulation and locomotor activity

After Trial 3 of the aggression test, the animal was moved to a small open field (460×80×303 mm acrylic cage; SCANET-40, MELQUEST, Toyama, Japan) with an optic fiber attached, and the locomotor activity was monitored for 10 min. One min of optical stimulation (3-ms pulses, 20 Hz) was delivered five times at 1-min intervals.

### Histology and microscopy

At the end of the experiment, mice were deeply anesthetized with sodium pentobarbital (Somnopentyl, Kyoritsu Seiyaku Co., Tokyo, Japan) and perfused intracardially with 0.9% saline followed by Zamboni solution (4% paraformaldehyde mixed with 0.2% picric acid in PBS). After post-fixation in Zamboni solution overnight, brains were placed into 30% sucrose solution. A cryostat was used to slice the brains into 40-µm sections, and the EYFP expression was examined under a fluorescent microscope (Zeiss AX10 Imager M1). For the c-Fos expression analysis, nine male mice that expressed either ChETA-EYFP (n = 5) or EYFP (n = 4) on excitatory neurons of the mPFC were used. Optical stimulation (20-Hz blue light, 3-ms pulses, 5 mW) was delivered to the mPFC for 1 min. Then, 1.5 hrs after the stimulation, the brain of the mice were fixed with Zamboni solution and 40-µm serial sections were prepared. Free-floating sections were first washed with PBS and then incubated with 3% normal goat serum (NGS) and 0.3% Triton-X in PBS for 1 hr at room temperature. Sections were incubated with primary antibody against c-Fos (rabbit anti-Fos (sc-52), 1∶8000, Santa Cruz Biotechnology, Santa Cruz, CA, USA) in PBS with 3% NGS and 0.3% Triton-X overnight at 4°C. After washing with PBS, the sections were incubated with anti-rabbit secondary antibody conjugated to the DyLight549 (1∶400, Jackson ImmunoResearch Laboratories, Inc., West Grove, PA, USA). Sections were then mounted on glass slides covered with gelatin, coverslipped with Fluoromount (Diagnostic BioSystems, Pleasanton, CA, USA) and observed by fluorescence microscopy (Zeiss AX10 Imager M1).

### Data analysis

Analysis of video-recorded resident–intruder encounters was conducted by a trained observer using free software (TanaMove0.07, http://www.nig.ac.jp/labs/MGRL/tanaMove.html) to quantify the frequency and duration of aggressive behaviors (attack bites, sideways threats, pursuit, and tail rattles) and nonaggressive behaviors (walking, rearing, self-grooming, and social contacts [22], [23]). The frequency of attack bites and durations of other behaviors were analyzed. A pairwise t-test with the Bonferroni correction was conducted to compare the effect of light stimulation between Trial 1 and Trial 2. For Trial 3, one-way repeated measures ANOVA was used to analyze to all sessions (three lights-on and three lights-off sessions). In the case of a significant *F* value, t-tests with the Bonferroni correction were conducted as *post hoc* analysis. The total number of attack bites during the lights-on and off periods was also calculated for Trial 3, and a pairwise t-test was then conducted to examine the effect of light stimulation.

Aggressive behavior probability was calculated by assessing the occurrence of either attack bites or sideways threats in each 0.05 sec time bin, and the number of incidence of aggressive behavior during each 10 second was divided by the occurrence of total aggressive behavior over a 12 min session. For aggressive burst analysis, when either attack bites or sideways threats occurred less than 1 sec apart, they were considered as part of a continuous burst. In this analysis, the frequency of bursts, the duration of bursts and the frequency of attack bites within one burst were examined during both the lights-on and lights-off periods. The attack/threat ratio was calculated by dividing the frequency of attack bites by the duration of sideways threat. The data from Trial 1, Trial 2 (lights-on), and Trial 3 were used for the mPFC–ChETA group, and data from Trial 1 and Trial 2 (lights-on) were used for the mPFC–eArchT3.0 group. Pairwise t-tests were conducted to examine the effect of light stimulation on the frequency of bursts, the frequency of attack bites within one burst, and the attack/threat ratio. For the duration of bursts, an unpaired t-test was conducted to compare all burst events during the lights-on and lights-off sessions. In addition, the distribution was summarized as a histogram based on the duration of each continuous burst. Pearson's chi-square test was used to compare the frequencies in the lights-on and lights-off periods in each class.

## Results

**Figure 1:**
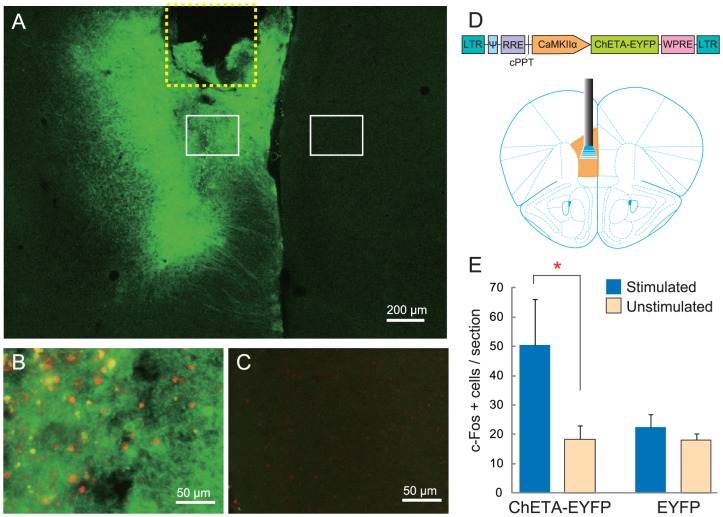
Injection of ChETA-EYFP lentivirus leads to functional ChETA expression on the excitatory neurons in the mPFC. In this study, the mPFC area includes both the prelimbic cortex and medial orbital cortex. (A) EYFP expression on one side of the mPFC. The yellow dotted line indicates the outline of the guide cannula, and white boxes indicate the areas of c-Fos analysis. (B) Increased c-Fos expression following 1-min of stimulation with blue light on the EYFP-expressing side of the mPFC. Green: EYFP, Red: cFos. (C) The contralateral side of the mPFC did not show an increase in c-Fos expression. (D) Schematic representations of the lentivirus construct and the site of mPFC activation. (E) Quantification of c-Fos-positive cells in the stimulated and contralateral unstimulated sides of the mPFC in ChETA-EYFP-expressing animals (n = 5) and control EYFP-expressing animals (n = 4). * p<0.05 by t-test. Error bars represent SEM.

To examine the inhibitory role of the mPFC in aggressive behavior, we used optogenetics to manipulate the activity of mPFC excitatory neurons during an aggressive encounter. Here, the prelimbic cortex (PrL) and medial orbital cortex (MO) are defined as parts of the mPFC area. By using Ca^2+^/calmodulin-dependent protein kinase α (CaMKIIα) promoter, we expressed the light-sensitive opsin ChETA (modified version of channelrhodopsin-2; ChR2 [18]) that was fused with the EYFP marker protein on the excitatory neurons within the mPFC. A guide cannula was stereotaxically inserted and the CaMKIIα::ChETA-EYFP lentivirus was microinjected into the unilateral mPFC (Figure 1A). All behavioral analyses with optical stimulation were conducted 3–6 weeks after the viral infection. Application of blue light (3-ms pulses, 20 Hz) for 1 min via optic fiber to the mPFC increased c-Fos expression in the stimulated side, but not in the other side, of the mPFC (Figure 1B–E). Control EYFP-expressing animals showed no difference in c-Fos expression between the stimulated side and the unstimulated side of the mPFC.

**Figure 2:**
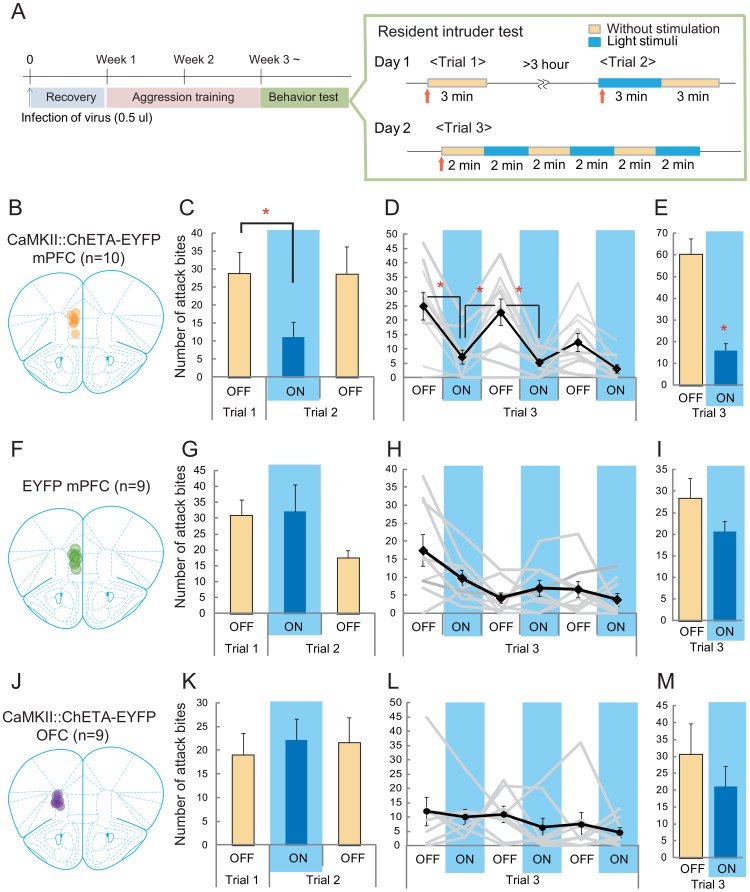
Activation of the mPFC, but not the OFC, inhibited attack bites of male mice. (A) Schematic representation of the test schedule. Lentivirus infection was carried out at least 3 weeks before the optical stimulation experiment. Trial 1 examined the resident male's basal aggressive behavior without light stimulation. In Trial 2, the light stimulus was delivered to the mPFC 5 sec before the introduction of the intruder, and continued during a 3-min aggressive encounter. The light stimulus was then removed, and aggressive behavior was observed for an additional 3 min. In Trial 3, 2-min light stimuli were delivered at intervals over a 12-min session. (B) Schematic diagram of the ChETA-EYFP expression and light stimulation in the mPFC. (C) During the lights-on period in Trial 2, there was a significant reduction of the number of attack bites compared with that in Trial 1. (D) Light stimuli presented at intervals inhibited the frequency of attack bites in Trial 3. (E) The total number of attack bites in Trial 3 was also significantly reduced during the lights-on period compared with that in the lights-off period. (F) Schematic diagram of the control EYFP expression and light stimulation in the mPFC. (G) There was no statistically significant difference between Trial 1 and Trial 2. (H) Light stimuli presented at intervals did not change the attack bite behavior in the control mice. (I) The total number of attack bites in Trial 3 did not differ between the lights-on and lights-off periods. (J) Schematic diagram of ChETA-EYFP expression and light stimulation in the OFC. In this study, the OFC area was defined as including both the ventral and lateral orbital cortex. (K) There was no statistically significant difference between Trial 1 and Trial 2. (L) Light stimuli presented at intervals did not affect attack bite behavior in the OFC-stimulated animals. (M) The total number of attack bites in Trial 3 did not differ between the lights-on and lights-off periods. * p<0.05 by t-test with Bonferroni correction. Error bars represent SEM.

**Figure 3:**
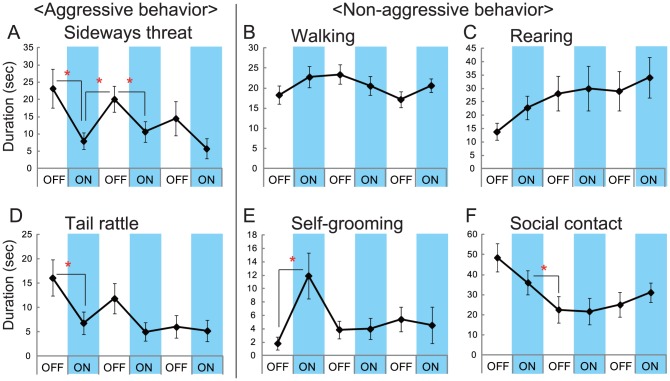
The mPFC activation inhibited aggressive behaviors specifically, and did not change non-aggressive behaviors in Trial 3. The images show the duration of sideways threat (A), walking (B), rearing (C), tail rattle (D), self-grooming (E) and social contact (F). Whereas (A) and (D) denote aggressive behavior, the other four behavior are non-aggressive behaviors. * p<0.05 by t-test with Bonferroni correction. Error bars represent SEM.

**Figure 4:**
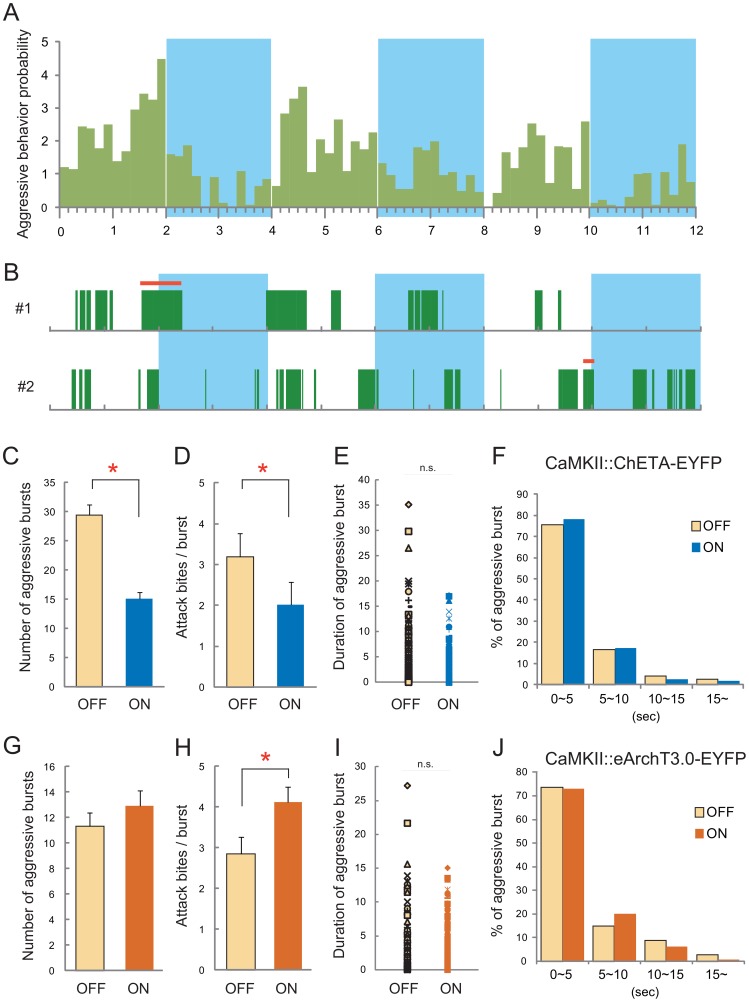
Aggressive burst analysis during activation and inhibition of the mPFC. (A) Temporal pattern of the probability of aggressive behavior during Trial 3 in ChETA expressing animals. Aggression probability was decreased throughout the light stimulation period. (B) Burst structure of aggressive behavior in individual ChETA expressing animals in Trial 3. The green bar indicates the occurrence of either attack bites or sideways threats. The red bar indicates an aggressive burst that started before the light stimulation and continued for a while after the light stimulation was applied. (C) The mPFC activation by ChETA stimulation caused a reduction of the frequency of aggressive bursts during the lights-on period. (D) The number of attack bites within a burst was lower during the lights-on period than during the lights-off period. (E) The duration of aggressive bursts was not changed by light stimulation in ChETA-expressing animals. (F) Analysis of the distribution of durations of aggressive bursts also showed no difference between the lights-on and lights-off periods. (G) Inhibition of the mPFC by eArchT3.0 increased the number of aggressive bursts slightly, albeit not significantly. (H) Inhibition of the mPFC increased the number of attack bites within a burst. (I–J) The duration of aggressive bursts was not changed by light stimulation in eArchT3.0-expressing animals. Whereas (C–F) were calculated by using the data from trial 1–3 in mPFC–ChETA group, (G–J) were calculated by using the data from trial 1 and trial 2 (lights-on) in mPFC–eArchT3.0 group. * p<0.05 by t-test. Error bars represent SEM.

We then examined aggressive behavior using the resident–intruder test. One week after the surgery, we started to expose the animals to situations in which aggressive behavior was exhibited every other day using the same intruder for 5 min until they showed more than ten attack bites over three consecutive encounters (Figure 2A). The animals were then tethered with an optic fiber at least 6 hrs before the first test, and an aggression test was conducted with optical stimulation. By activating the mPFC excitatory neurons (Figure 2B and Figure S1), we found that the animals showed lower level of attack bites during the lights-on session (Trial 2) than during the lights-off session (Trial 1) (t(9) = 3.860, p = 0.0076; Figure 2C). After the light stimulus had ceased (Trial 2 lights-off), the number of attack bites returned to the same level as in Trial 1. This inhibitory effect of light stimulation was confirmed by the alternative presentations of 2-min periods of lights-on and lights-off exposure during a 12-min aggressive encounter (Trial 3; Figure 2D and Movie S1), and activation of the mPFC reduced the number of attack bites (F(9,45) = 8.566, p<0.0001). A significant reduction in the number of attack bites was observed until the second lights-on session, and there was a significant effect of light stimulation on the total number of attack bites in Trial 3 (t(9) = 7.338, p<0.0001; Figure 2E). A similar pattern was observed in other aggressive behaviors (Figure 3), and significant reductions of the duration of sideways threat (F(9,45) = 3.843, p = 0.0055) and tail rattle (F(9,45) = 4.109, p = 0.0037) were also observed during the lights-on sessions compared with those during the lights-off sessions (Figure 3A,D, Table S1). The temporal pattern of aggressive behavior during Trial 3 showed that the probability of such behavior was decreased throughout the period of light stimulation (Figure 4A). On the other hand, light had no effect on non-aggressive behaviors during Trial 1 and 2 (Table S1) or open-field activity (Figure S2). One-way repeated measures ANOVA of the data collected during Trial 3 showed that there were significant effects of session on rearing, self-grooming, and social contact (F(9,45)≤0.0048, p<0.005). However, over the 12 min trial, consistent increases and decreases in temporal patterns of rearing and social contact, respectively, suggested that these behavioral changes resulted from habituation to the test situation (Figure 3C,F). In contrast, whereas self-grooming was significantly increased during the first lights-on session of Trial 3 (Figure 3E), no such increase in self-grooming was observed during Trial 2 (Table S1). Light stimulus had no effect on either aggressive or non-aggressive behaviors in the animals infected with the control virus (EFα::EYFP or CaMKIIα::EYFP, Figure 2F–I, Figure S3).

Aggressive behaviors have a burst-like temporal pattern, with the animal engaging in such behaviors for a certain period in a cluster (aggressive burst), followed by a rest period during which the animal shows non-aggressive exploratory behaviors [24]. During the analysis of the ChETA-expressing mPFC subjected to optical stimulation, we found that the effect of the mPFC activation on aggressive behavior was not very acute; when an animal engaged in an aggressive burst before the light stimulation, the burst continued for a while after the beginning of the light stimulation (Figure 4B, red lines). To understand the details of the effect of mPFC activation on the temporal pattern of aggressive behavior, we examined the burst structure of the aggressive behavior during the lights-on and lights-off periods. We defined a continuous burst when either attack bites or sideways threats occurred less than 1 sec apart. Burst analysis showed that activation of the mPFC reduced both the number of aggressive bursts (t(9) = 6.257, p = 0.0001), and the frequency of attack bites within a burst (t(9) = 3.136, p = 0.0120) compared with the levels of both during the lights-off period (Figure 4C,D). On the other hand, light stimulation did not change the duration of aggressive bursts (t(288) = 1.280, p = 0.2012; Figure 4E). A histogram also showed there was no difference in the duration of aggressive bursts in any class (Figure 4F). Therefore, the mPFC suppresses the probability of aggressive behaviors, but does not affect the termination of aggressive bursts.

**Figure 5:**
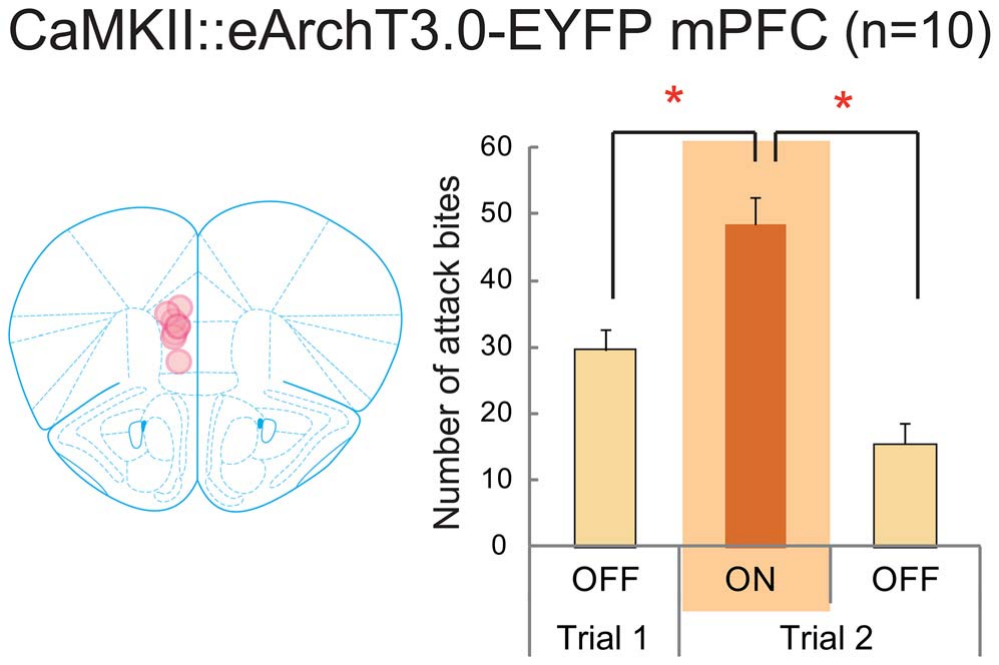
Inhibition of the mPFC activity by the expression of eArchT3.0 on the excitatory neurons of the mPFC. *(Left)* Schematic diagram of eArchT3.0 expression and light stimulation in the mPFC. *(Right)* During the lights-on period in Trial 2, there was a significant increase in the number of attack bites compared with that in the lights-off period in Trial 1 and Trial 2. * p<0.05 by t-test with Bonferroni correction. Error bars represent SEM.

To confirm the inhibitory role of the mPFC in aggressive behavior, we aimed to inhibit mPFC activity by expressing archaerhodopsin (eArchT3.0 [17]) on excitatory neurons in the mPFC by infection with CaMKIIα::eArchT3.0-EYFP lentivirus. Owing to the possible long-lasting inhibitory effect of eArch3.0 on the neurons, even after light termination [25], we performed only Trials 1 and 2 but not Trial 3, as defined above, in this experiment. We found that a lights-on session (Trial 2) resulted in a significantly higher number of attack bites than a lights-off session (Trial 1) (t(9) = −4.429, p = 0.0032; Figure 5 and Figure S4). After the light stimulus ceased (Trial 2 lights-off), the number of attack bites was significantly reduced compared with the level in the lights-on period (t(9) = 13.165, p<0.0001). However, there was no significant effect of light stimulation on other aggressive or non-aggressive behaviors when a lights-on session (Trial 2) was compared with a lights-off session (Trial 1) (Table S1). We also analyzed the burst structure in the animals that expressed eArchT3.0 in the mPFC. Again, there was no effect of light stimulation on the duration of aggressive bursts (t(240) = 0.507, p = 0.6127; Figure 4I, J). On the other hand, optogenetic silencing of the mPFC significantly increased the frequency of attack bites within a burst compared with the level during the lights-off period (t(9) = −2.410, p = 0.0392; Figure 4H). These results suggest that mPFC activation changes the intensity of the aggressive bursts.

**Figure 6:**
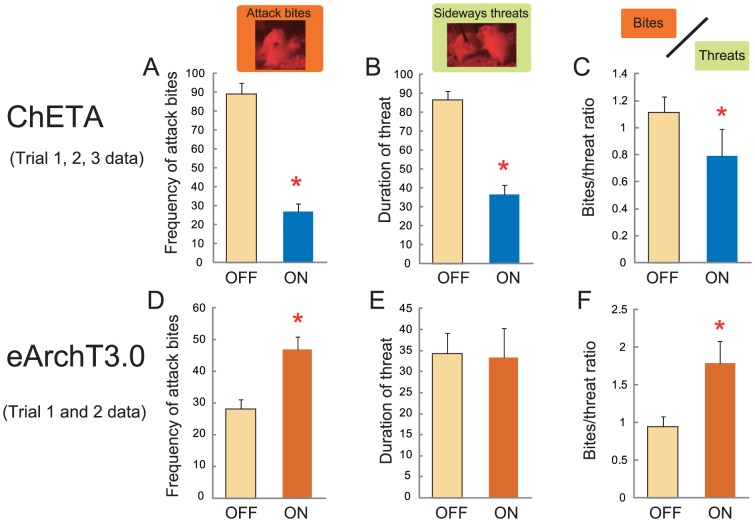
Effect of the mPFC activation and inhibition on the attack and threat ratio of aggressive behavior. (A) All data from Trials 1–3 were combined to estimate the attack-threat ratio for the ChETA experiment. There were significant reductions of the number of attack bites (A) and the duration of sideways threats (B) after mPFC activation. (C) There was a significant reduction of the attack/threat ratio by light stimulation. Inhibition of the mPFC by eArchT3.0 stimulation increased the frequency of attack bites (D), but had no effect on the duration of sideways threats (E). (F) There was a significant increase in the attack/threat ratio caused by stimulation. * p<0.05 by t-test. Error bars represent SEM.

To examine the characteristics of the aggressive behavior further, we examined the ratio of the numbers of physical attacks (bites) to threatening behaviors [26], [27] during mPFC activation and inhibition. In the ChETA-expressing animals, activation of the mPFC reduced both the number of attack bites and the duration of sideways threats, and there was a significant reduction in the bite/threat ratio during the lights-on period (t(9) = 3.596, p = 0.0058; Figure 6A–C). At the same time, inhibition of the mPFC by eArchT3.0 increased the number of attack bites, but not the sideways threats, and thus there was a significant increase of attack/threat ratio during the lights-on period (t(9) = −2.822, p = 0.0200; Figure 6D–F). There were thus more physical attacks than incidents of threatening behavior during the lights-on period in eArchT3.0-expressing animals.

To examine the role of OFC activity in the aggressive behavior of male mice, we expressed ChETA-EYFP on excitatory neurons in the OFC of male mice, and activated those neurons during an aggressive encounter. Here, the ventral orbital cortex (VO) and lateral orbital cortex (LO) were included in the definition of the OFC area. In contrast to mPFC, there was no clear effect of OFC stimulation on either aggressive or non-aggressive behaviors (Figure 2J–M, Figure S3, Table S1).

## Discussion

Although there is consensus regarding the involvement of the prefrontal cortex in aggression and violence in a wide range of animals [1], [2], it remains unclear which phase of aggressive behavior (initiation, execution and termination) is modulated by prefrontal activity. We have herein provided direct evidence for an inhibitory role of mPFC activity on the initiation and execution of aggressive behavior. Activation of the mPFC by light opsin ChETA expressed on excitatory neurons specifically inhibited aggressive behaviors (attack bites, sideways threats and tail rattles), without affecting locomotor activity or the social contact behavior of male mice during resident–intruder encounters. Lin *et al*. (2011) showed that optical activation of the hypothalamic VMHvl (the ventromedial hypothalamus, ventrolateral subdivision) in male mice induced attack bites toward any opponent, including females and even inanimate objects. This effect was very direct and acute: the application of a light pulse into the VMHvl immediately induced attack bites, and this behavior stopped immediately after the termination of the light stimulus [28]. On the other hand, we found that activation of the mPFC has a rather modulatory effect on aggressive behavior. The activation of the mPFC did not stop an aggressive burst immediately, but instead reduced the total probability of the occurrence of aggressive behavior. We also found that the intensity of aggressive behaviors changed to a more intense form of aggression by the inhibition of mPFC activity. The silencing of mPFC activity by eArchT3.0 caused an increase in the number of attack bites during aggressive bursts, and showed an escalated form of behavioral pattern in which animals engaged more in physical attacks (biting) than in threatening behavior. Thus, normal activity of the mPFC is required to maintain the level of aggressive behavior within the species-typical range. By contrast, our results showed that neither activation nor inhibition of the mPFC activity changed the duration of the aggressive bursts. Therefore, mPFC activation cannot terminate an aggressive burst once it has started.

Yizhar *et al*. (2011) reported that activation of the mPFC by CaMKIIα::SSFO (ChR2(C128S/D156A)) caused a strong reduction in social affiliative behaviors. It is therefore possible that, in our study, mPFC activation reduced social interest which in turn reduced aggressive behavior. However, light stimulation during the resident–intruder test caused a slight increase in social contact rather than a reduction in social contact (Table S1). This discrepancy between studies might have been caused by the difference in the activation frequency between SSFO (around 80 Hz [29]) and the activation frequency used in our study (20 Hz). We therefore also attempted to activate our ChETA receptors using a frequency of 80 Hz (3-msec pulses). However, our preliminary result indicated that a higher frequency of activation of the mPFC excitatory neurons during the resident–intruder test did not reduce the extent of social contact (Figure S5H). Therefore, the activation of excitatory neurons in the mPFC specifically inhibited aggressive behaviors in adult male mice that had been exposed to winning experiences.

It is likely that the mPFC inhibits activity of a neural circuit that is involved in the execution of aggressive behaviors. In fact, the mPFC sends glutamatergic projections to brain areas that have been implicated in aggressive behaviors [3], [4], [30], [31], such as the anterior hypothalamus [32], [33], medial amygdala [34], ventral tegmental area (VTA) [35] and DRN [36], [37]. It has been shown that activation of the mPFC inhibits the activity of DRN serotonergic neurons [38]–[40]. A greater population of mPFC glutamatergic neurons extended their projections to the GABA interneurons more than 5-HT neurons in the DRN [41], and thus mPFC activation increases GABA input within the DRN [42]. Similarly, the mPFC extends projections to a set of GABA neurons in the VTA that in turn project to the nucleus accumbens [43]. Thus, it is likely that mPFC activation can inhibit some of the neural pathways that constitute the aggression circuit. By contrast, it seems that the VMHvl, which has been strongly implicated in aggressive behavior [28], [44], [45], does not receive direct input from the mPFC [4], [46]. Further study is required to dissect the specific mPFC projections that control escalated aggression.

In contrast to lesion or pharmacological studies, the current study did not find any effect of OFC activation on aggressive behavior. Pharmacological studies have shown that the activation of 5-HT_1A_ or 5-HT_1B_ receptors by agonist microinjection into this area reduces aggressive behavior [47], [48]. However, the sites of action of the 5-HT agonists are not only glutamatergic neurons but also other types of neurons (e.g. GABAergic neurons) in the prefrontal cortex [49]. Thus it is not clear how the OFC glutamatergic neurons were manipulated by the administration of an aggression-suppressive dose of 5-HT agonist. Lesion studies in rats have shown that a lack of OFC increased the incidence of aggressive behavior [9], [10]. Therefore, in combination with our findings, it is likely that the activity of OFC excitatory neurons is required, but not sufficient, to inhibit intermale aggression.

## Supporting Information

Figure S1 Representative picture of EYFP expression on the unilateral side of the mPFC or OFC. (A) ChETA-EYFP expression in the mPFC. (B) eArchT3.0-EYFP expression in the mPFC. (C) EYFP control expression in the mPFC. (D) ChETA-EYFP expression in the OFC.
(EPS)

Figure S2 Locomotor activity in the open field was not changed by light stimulation of the mPFC or OFC.
(EPS)

Figure S3 Aggressive behaviors and non-aggressive behaviors in EYFP-expressing control animals (top panel) and animals with ChETA expression in the OFC (bottom panel).
(EPS)

Figure S4 Temporal pattern of the attack bites in eArchT3.0-expressing animals. The number of attack bites was increased in each individual in Trial 2 (B) compared with those in Trial 1 (A). (C) Cumulative number of attack bites indicates that animals without stimulation showed high level of attack bites during first minute and decrease later session. (D) eArchT3.0 activation caused high level of attack bites throughout the three-min trial.
(EPS)

Figure S5 Activation of the ChETA in the mPFC by using 80-Hz blue light stimulation (n = 4). Although no statistical significance was identified, there were trends of reductions of aggressive behaviors (A–D) and self-grooming (G) by light stimulation. On the other hand, there was no effect of light stimulation on walking (E) and rearing (F). In addition, we did not observe any reduction of social contact by the mPFC activation (H).
(EPS)

Movie S1 Activation of the ChETA in the mPFC inhibited attack bites of male mice in the resident–intruder test (Trial 3).
(AVI)

Table S1 Aggressive and non-aggressive behaviors in Trial 1 and Trial 2. Pairwise t-test was conducted to compare Trial 1 (lights-off) vs. Trial 2 (lights-on), or lights-on vs. off within Trial 2 (* p<0.05).
(DOCX)

## References

[1] Nelson RJ, Trainor BC (2007) Neural mechanisms of aggression. Nat. Rev. Neurosci 8: 536–546. 17585306 10.1038/nrn2174

[2] Davidson RJ, Putnam KM, Larson CL (2000) Dysfunction in the Neural Circuitry of Emotion Regulation–A Possible Prelude to Violence. Science 289: 591–594. 10915615 10.1126/science.289.5479.591

[3] Hoover WB, Vertes RP (2011) Projections of the medial orbital and ventral orbital cortex in the rat. J. Comp. Neurol 519: 3766–3801. 21800317 10.1002/cne.22733

[4] Vertes RP (2004) Differential projections of the infralimbic and prelimbic cortex in the rat. Synapse 51: 32–58. 14579424 10.1002/syn.10279

[5] Gabbott PLA, Warner TA, Jays PRL, Salway P, Busby SJ (2005) Prefrontal cortex in the rat: projections to subcortical autonomic, motor, and limbic centers. J. Comp. Neurol 492: 145–177. 16196030 10.1002/cne.20738

[6] Brower MC, Price BH (2001) Neuropsychiatry of frontal lobe dysfunction in violent and criminal behaviour: a critical review. J. Neurol. Neurosurg. Psychiatry 71: 720–726. 11723190 10.1136/jnnp.71.6.720 PMC1737651

[7] Raine A, Buchsbaum M, LaCasse L (1997) Brain abnormalities in murderers indicated by positron emission tomography. Biol. Psychiatry 42: 495–508. 10.1016/S0006-3223(96)00362-9 9285085

[8] An X, Bandler R, Ongür D, Price JL (1998) Prefrontal cortical projections to longitudinal columns in the midbrain periaqueductal gray in macaque monkeys. J. Comp. Neurol 401: 455–479. 9826273

[9] de Bruin JP, van Oyen HG, Van de Poll N (1983) Behavioural changes following lesions of the orbital prefrontal cortex in male rats. Behav. Brain Res 10: 209–232. 6686460 10.1016/0166-4328(83)90032-3

[10] Kolb B, Nonneman AJ (1974) Frontolimbic lesions and social behavior in the rat. Physiol. Behav 13: 637–643. 10.1016/0031-9384(74)90234-0 4610602

[11] Halász J, Tóth M, Kalló I, Liposits Z, Haller J (2006) The activation of prefrontal cortical neurons in aggression–a double labeling study. Behav. Brain Res 175: 166–175. 16978716 10.1016/j.bbr.2006.08.019

[12] Haller J, Tóth M, Halasz J, de Boer SF (2006) Patterns of violent aggression-induced brain c-fos expression in male mice selected for aggressiveness. Physiol. Behav 88: 173–182. 16687160 10.1016/j.physbeh.2006.03.030

[13] Wall VK, Fischer EK, Bland ST (2012) Isolation rearing attenuates social interaction-induced expression of immediate early gene protein products in the medial prefrontal cortex of male and female rats. Physiol. Behav 107: 440–450. 22982514 10.1016/j.physbeh.2012.09.002 PMC4529065

[14] Wang F, Zhu J, Zhu H, Zhang Q, Lin Z, et al (2011) Bidirectional control of social hierarchy by synaptic efficacy in medial prefrontal cortex. Science 334: 693–697. 21960531 10.1126/science.1209951

[15] Siegel A, Edinger H, Dotto M (1975) Effects of electrical stimulation of the lateral aspect of the prefrontal cortex upon attack behavior in cats. Brain Res. 93: 473–484. 10.1016/0006-8993(75)90185-7 1236761

[16] Siegel A, Edinger H, Lowenthal H (1974) Effects of electrical stimulation of the medial aspect of the prefrontal cortex upon attack behavior in cats. Brain Res. 66: 467–479. 10.1016/0006-8993(75)90185-7 1236761

[17] Mattis J, Tye KM, Ferenczi EA, Ramakrishnan C, O'Shea DJ, et al (2012) Principles for applying optogenetic tools derived from direct comparative analysis of microbial opsins. Nat. Methods 9: 159–172. 10.1038/nmeth.1808 PMC4165888 22179551

[18] Gunaydin LA, Yizhar O, Berndt A, Sohal VS, Deisseroth K, et al (2010) Ultrafast optogenetic control. Nat. Neurosci 13: 387–392. 20081849 10.1038/nn.2495

[19] Hewinson J, Paton JFR, Kasparov S (2013) Viral gene delivery: optimized protocol for production of high titer lentiviral vectors. Methods Mol. Biol 998: 65–75. 23529421 10.1007/978-1-62703-351-0_5

[20] Franklin K, Paxinos G (2008) The mouse brain in stereotaxic coordinates. Third edit. San Diego: Academic Press.

[21] Golden SA, Covington HE 3rd, Berton O, Russo SJ (2011) A standardized protocol for repeated social defeat stress in mice. Nat. Protoc 6: 1183–1191. 21799487 10.1038/nprot.2011.361 PMC3220278

[22] Grant EC, Mackintosh JH (1963) A comparison of the social postures of some common laboratory rodents. Behaviour 21: 246–259.

[23] Miczek KA, O'Donnell JM (1978) Intruder-evoked aggression in isolated and nonisolated mice: effects of psychomotor stimulants and L-dopa. Psychopharmacology 57: 47–55. 26933 10.1007/BF00426957

[24] Miczek KA, Weerts EM, Tornatzky W, DeBold JF, Vatne TM (1992) Alcohol and “bursts” of aggressive behavior: ethological analysis of individual differences in rats. Psychopharmacology 107: 551–563. 1603899 10.1007/BF02245270

[25] Tsunematsu T, Tabuchi S, Tanaka KF, Boyden ES, Tominaga M, et al (2013) Long-lasting silencing of orexin/hypocretin neurons using archaerhodopsin induces slow-wave sleep in mice. Behav. Brain Res 255: 64–74. 23707248 10.1016/j.bbr.2013.05.021

[26] Haller J, Kruk MR (2006) Normal and abnormal aggression: human disorders and novel laboratory models. Neurosci. Biobehav. Rev 30: 292–303. 16483889 10.1016/j.neubiorev.2005.01.005

[27] Miczek KA, de Boer SF, Haller J (2013) Excessive aggression as model of violence: a critical evaluation of current preclinical methods. Psychopharmacology 226: 445–458. 23430160 10.1007/s00213-013-3008-x PMC3595336

[28] Lin D, Boyle MP, Dollar P, Lee H, Lein ES, et al (2011) Functional identification of an aggression locus in the mouse hypothalamus. Nature 470: 221–226. 21307935 10.1038/nature09736 PMC3075820

[29] Yizhar O, Fenno LE, Prigge M, Schneider F, Davidson TJ, et al (2011) Neocortical excitation/inhibition balance in information processing and social dysfunction. Nature 477: 171–178. 21796121 10.1038/nature10360 PMC4155501

[30] Rosenkranz JA, Grace AA (2002) Cellular mechanisms of infralimbic and prelimbic prefrontal cortical inhibition and dopaminergic modulation of basolateral amygdala neurons in vivo. J. Neurosci 22: 324–337. 11756516 10.1523/JNEUROSCI.22-01-00324.2002 PMC6757602

[31] Peyron C, Petit JM, Rampon C, Jouvet M, Luppi PH (1997) Forebrain afferents to the rat dorsal raphe nucleus demonstrated by retrograde and anterograde tracing methods. Neuroscience 82: 443–468. 10.1016/s0306-4522(97)00268-6 9466453

[32] Gobrogge KL, Liu Y, Young LJ, Wang Z (2009) Anterior hypothalamic vasopressin regulates pair-bonding and drug-induced aggression in a monogamous rodent. Proc. Natl. Acad. Sci. U. S. A. 106: 19144–19149. 19858480 10.1073/pnas.0908620106 PMC2776424

[33] Ferris CF, Melloni RH Jr, Koppel G, Perry KW, Fuller RW, et al (1997) Vasopressin/serotonin interactions in the anterior hypothalamus control aggressive behavior in golden hamsters. J. Neurosci 17: 4331–4340. 9151749 10.1523/JNEUROSCI.17-11-04331.1997 PMC6573530

[34] Koolhaas JM, Van Den Brink THC, Roozendaal B, Boorsma F (1990) Medial amygdala and aggressive behavior: Interaction between testosterone and vasopressin. Aggress. Behav 16: 223–229.

[35] Fuxjager MJ, Forbes-Lorman RM, Coss DJ, Auger CJ, Auger AP, et al (2010) Winning territorial disputes selectively enhances androgen sensitivity in neural pathways related to motivation and social aggression. Proc. Natl. Acad. Sci 107: 12393–12398. 20616093 10.1073/pnas.1001394107 PMC2901436

[36] Takahashi A, Shimamoto A, Boyson CO, DeBold JF, Miczek KA (2010) GABA_B_ receptor modulation of serotonin neurons in the dorsal raphe nucleus and escalation of aggression in mice. J. Neurosci 30: 11771–11780. 20810897 10.1523/JNEUROSCI.1814-10.2010 PMC2943331

[37] Faccidomo S, Bannai M, Miczek KA (2008) Escalated aggression after alcohol drinking in male mice: dorsal raphé and prefrontal cortex serotonin and 5-HT_1B_ receptors. Neuropsychopharmacology 33: 2888–2899. 18305458 10.1038/npp.2008.7

[38] Celada P, Puig MV, Casanovas JM, Guillazo G, Artigas F (2001) Control of dorsal raphe serotonergic neurons by the medial prefrontal cortex: Involvement of serotonin-1A, GABA_A_, and glutamate receptors. J. Neurosci 21: 9917–9929. 11739599 10.1523/JNEUROSCI.21-24-09917.2001 PMC6763042

[39] Hajós M, Richards CD, Székely AD, Sharp T (1998) An electrophysiological and neuroanatomical study of the medial prefrontal cortical projection to the midbrain raphe nuclei in the rat. Neuroscience 87: 95–108. 9722144 10.1016/s0306-4522(98)00157-2

[40] Varga V, Kocsis B, Sharp T (2003) Electrophysiological evidence for convergence of inputs from the medial prefrontal cortex and lateral habenula on single neurons in the dorsal raphe nucleus. Eur. J. Neurosci 17: 280–286. 12542664 10.1046/j.1460-9568.2003.02465.x

[41] Jankowski MP, Sesack SR (2004) Prefrontal cortical projections to the rat dorsal raphe nucleus: ultrastructural features and associations with serotonin and gamma-aminobutyric acid neurons. J. Comp. Neurol 468: 518–529. 14689484 10.1002/cne.10976

[42] Challis C, Boulden J, Veerakumar A, Espallergues J, Vassoler FM, et al (2013) Raphe GABAergic neurons mediate the acquisition of avoidance after social defeat. J. Neurosci 33: 13978–13988. 23986235 10.1523/JNEUROSCI.2383-13.2013 PMC3756748

[43] Carr DB, Sesack SR (2000) Projections from the rat prefrontal cortex to the ventral tegmental area: target specificity in the synaptic associations with mesoaccumbens and mesocortical neurons. J. Neurosci 20: 3864–3873. 10804226 10.1523/JNEUROSCI.20-10-03864.2000 PMC6772693

[44] Yang CF, Chiang MC, Gray DC, Prabhakaran M, Alvarado M, et al (2013) Sexually dimorphic neurons in the ventromedial hypothalamus govern mating in both sexes and aggression in males. Cell 153: 896–909. 23663785 10.1016/j.cell.2013.04.017 PMC3767768

[45] Sano K, Tsuda MC, Musatov S, Sakamoto T, Ogawa S (2013) Differential effects of site-specific knockdown of estrogen receptor α in the medial amygdala, medial pre-optic area, and ventromedial nucleus of the hypothalamus on sexual and aggressive behavior of male mice. Eur. J. Neurosci 37: 1308–1319. 23347260 10.1111/ejn.12131

[46] Floyd NS, Price JL, Ferry AT, Keay KA, Bandler R (2001) Orbitomedial prefrontal cortical projections to hypothalamus in the rat. J. Comp. Neurol 432: 307–328. 11246210 10.1002/cne.1105

[47] Centenaro LA, Vieira K, Zimmermann N, Miczek KA, Lucion AB, et al (2008) Social instigation and aggressive behavior in mice: role of 5-HT_1A_ and 5-HT_1B_ receptors in the prefrontal cortex. Psychopharmacology 201: 237–248. 18688602 10.1007/s00213-008-1269-6 PMC4371733

[48] de Almeida RMM, Rosa MM, Santos DM, Saft DM, Benini Q, et al (2006) 5-HT_1B_ receptors, ventral orbitofrontal cortex, and aggressive behavior in mice. Psychopharmacology 185: 441–150. 16550387 10.1007/s00213-006-0333-3

[49] Andrade R (2011) Serotonergic regulation of neuronal excitability in the prefrontal cortex. Neuropharmacology 61: 382–386. 21251917 10.1016/j.neuropharm.2011.01.015 PMC3110517

