# Supplementary material for: Control of Intermale Aggression by Medial Prefrontal Cortex Activation in the Mouse
Source: PLoS One. 2014 Apr 16;9(4):e94657. doi: 10.1371/journal.pone.0094657 (PMC3989250; doi:10.1371/journal.pone.0094657)
Supplement: Table S1 — Aggressive and non-aggressive behaviors in Trial 1 and Trial 2. Pairwise t-test was conducted to compare Trial 1 (lights-off) vs. Trial 2 (lights-on), or lights-on vs. off within Trial 2 (* p<0.05). (DOCX) [file pone.0094657.s007.docx]

Table S1. Aggressive and non-aggressive behaviors in Trial 1 and Trial 2


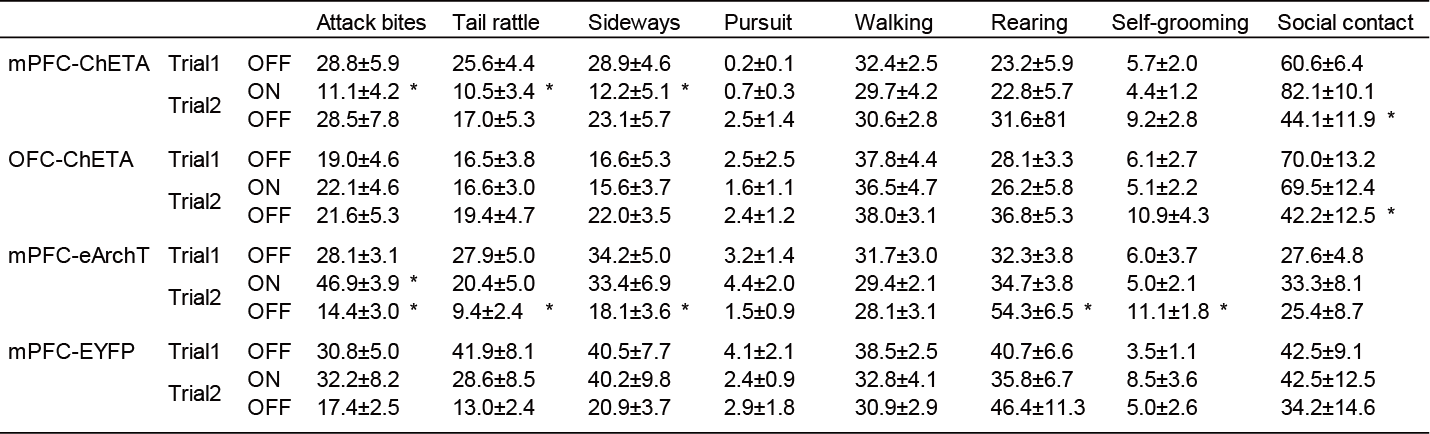


Pairwise t-test to compare Trial 1 (lights-off) vs. Trial 2 (lights-on), or lights-on vs. off within Trial 2 (* p < 0.05)
